# Symptom assessment after nasal irrigation with xylitol in the postoperative period of endonasal endoscopic surgery

**DOI:** 10.1016/j.bjorl.2020.05.023

**Published:** 2020-07-07

**Authors:** Caroline Feliz Fonseca Sepeda da Silva, Flávia Emilly Rodrigues da Silva, Henrique Furlan Pauna, Johann Gustavo Guilhermo Melcherts Hurtado, Marco Cesar Jorge dos Santos

**Affiliations:** a Instituto Paranaense de Otorrinolaringologia (IPO), Curitiba, PR, Brazil; b Pontifícia Universidade Católica do Paraná (PUC-PR), Curitiba, PR, Brazil; c Universidade de São Paulo (FMRP-USP), Faculdade de Medicina de Ribeirão Preto, Ribeirão Preto, SP, Brazil; d Universidade Federal do Paraná, Hospital de Clínicas, Curitiba, PR, Brazil

**Keywords:** Sinusitis, Nasal polyps, Therapeutic irrigation, Xylitol, Endoscopy

## Abstract

**Introduction:**

Chronic rhinosinusitis is an inflammatory condition of the nasal cavity and the paranasal sinuses that requires multifactorial treatment. Xylitol can be employed with nasal irrigation and can provide better control of the disease.

**Objective:**

To evaluate the association between the effects of nasal lavage with saline solution compared to nasal lavage with a xylitol solution.

**Methods:**

Fifty-two patients, divided into two groups (n = 26 in the “Xylitol” group and n = 26 in the “Saline solution” group) answered questionnaires validated in Portuguese (NOSE and SNOT-22) about their nasal symptoms and general symptoms, before and after endonasal endoscopic surgery and after a period of 30 days of nasal irrigation.

**Results:**

The “Xylitol” group showed significant improvement in pain relief and nasal symptom reduction after surgery and nasal irrigation with xylitol solution (*p* < 0.001). The “Saline solution” group also showed symptom improvement, but on a smaller scale.

**Conclusion:**

This study suggests that the xylitol solution can be useful in the postoperative period after endonasal endoscopic surgery, because it leads to a greater reduction in nasal symptoms.

## Introduction

Chronic rhinosinusitis (CRS) is an inflammatory condition of the nasal cavity mucosa and paranasal sinuses with symptoms lasting longer than 12 weeks.^1^ It can be divided into primary or secondary chronic rhinosinusitis (with or without nasal polyposis). Polyps are the final stage of hyperplastic growth of the nasal mucosa due to the intense activity of type 2 T-helper lymphocytes (Th2), eosinophils and Immunoglobulin-E (IgE).^1^

In chronic sinusitis the bacterial presence interferes with mucociliary clearance, innate and cell immunity.^2^ The relationship between CRS and biofilms was described in 2004 and several therapies have been proposed to eradicate these entities, aiming for adequate symptom control and reduction in disease severity.^3^ Among the several currently recommended treatments, we highlight: nasal corticoids, implants with intranasal corticoids, systemic corticoids, and nasal lavage with saline solution and immunotherapy.^1^

Xylitol (1,2,3,4,5-pentahydroxypentane) is an open-chain polyalcohol with five hydroxyl groups, nontoxic and safe, according to the Food and Drug Administration.^4^ It has a sweetening power similar to sucrose – with 40% less calories – and is metabolized by the liver into glucose and glycogen or pyruvate and lactate.^5^ It is well tolerated, although there are no established maximum doses. Nasal irrigation is commonly recommended, both in the pre- and postoperative periods, to improve mucociliary clearance, decrease edema, reduce the concentration of inflammatory mediators and reduce the amount of mucus accumulated in the cavities, in addition to preventing the formation of crusts.^6^ Recent studies have demonstrated the effectiveness of xylitol associated with nasal irrigation during the postoperative period,^6^ due to its antibacterial, bactericidal and anti-adhesive characteristics demonstrated *in vitro*.^7^

Xylitol use has several therapeutic purposes and the most established ones are the prevention of cavities^8^ and stabilization of glycemic levels, as it does not depend on insulin to be metabolized.^9^ In the respiratory system, xylitol can provide the following therapeutic effects:

a) Bacterial toxicity: The xylitol molecule is not metabolized by the bacteria and has to be eliminated from the cell. This promotes a cycle of energy expenditure without nutritional gain which, together with the toxic accumulation of xylitol phosphate inside the cell, triggers bacterial death^10^;
b) Bacterial adhesion: Extracellular xylitol acts as an analog receptor for the host cell, interfering with the adhesion process^11^, ^12^;
c) Increase in the innate defenses of the airway surface: The antibacterial activity of several of these agents is salt-dependent, that is, an increase in their concentration will inhibit their performance, both alone and synergistically. Some authors believe that this change in concentration is one of the reasons why patients with cystic fibrosis are more prone to bacterial colonization and infection^13^;
d) Nitric oxide: Nitric oxide produced by activated macrophages is important for the antimicrobial immune response.^14^ It has been demonstrated that 5% xylitol stimulates the production of nitric oxide by macrophages infected with *Leishmania amazonenses*, decreasing the infection after 72 h^15^;
e) Biofilm: Xylitol has shown to be effective in inhibiting the formation of bacterial biofilm in the oral cavity, mainly in studies related to the formation of plaque and cavities.^16^

Previous clinical studies have demonstrated the effectiveness of xylitol in reducing nasal symptoms in patients without associated surgery (through the visual analog scale and the SNOT-22 questionnaire).^17^, ^18^ However, how much of its concentration and volume is needed to reduce nasal symptoms in the postoperative period of Functional Endoscopic Sinus Surgery (FESS) and the comparison of the efficacy between this type of nasal lavage and saline lavage is still not well established.

Therefore, we question whether the use of the xylitol solution shows superiority when compared to the traditional 0.9% physiological saline solution for nasal irrigation in the postoperative period of patients submitted to endoscopic paranasal sinus surgery. In an attempt to answer this question, the present study aims to comparatively analyze, through clinical parameters, the effectiveness of large volume nasal irrigation with xylitol solution and 0.9% saline solution in the postoperative period of patients undergoing endoscopic paranasal sinus surgery.

## Methods

### Population

Fifty-two patients diagnosed with CRS (primary or secondary, with or without nasal polyposis), who met the criteria of difficult-to-treat CRS, that is, those who did not achieve adequate clinical control after using topical nasal spray corticosteroids and up to two cycles of antibiotics and/or oral corticosteroids in the last year, followed at a specialized outpatient clinic, submitted to endonasal endoscopic surgery, performed by the same surgeon, and using the same technique to open all paranasal sinuses.

The diagnosis of CRS was defined according to the research criteria suggested by EPOS 2020.^1^ Patients under 18 years old or who did not wish to participate in the study were excluded. The study was approved by the Institution's Research Ethics Committee under number 266/2019.

### Study design

Prospective uncontrolled intervention study in patients with difficult-to-treat CRS. The study intervention consisted of topical therapy, with high volume nasal irrigation with xylitol solution. Patients were instructed to start nasal irrigation (with xylitol or saline) beginning the second postoperative day.

The patients in the “Xylitol” group were instructed to use the solution as follows: each sachet of 6 g of xylitol was to be diluted in 360 mL of filtered or boiled water. The patients were instructed to use this volume of 360 mL per day, irrigating each nostril with the aid of a 60 mL syringe, with a continuous jet of the solution every eight hours, during a period of thirty days.

As for the patients in the “Saline” group, they were instructed to use 360 mL of 0.9% saline solution daily by irrigating the nasal cavities with the aid of a 60 mL syringe, using the saline solution in each nostril every eight hours during a period of 30 days. Table 1 shows the budget values for the present study.

**Table 1:** Budget values for this study.

Identification	Cost (R$)
Xylitol (14.4 kg)	720.00
60 mL syringe (100 units)	500.00
0.9% saline solution (540 L)	2700.00

Patients were evaluated before and after 30 days of topical irrigation therapy. The outcomes assessed were: subjective improvement and degree of satisfaction after topical irrigation therapy. Patients were asked, at the end of the topical irrigation therapy, whether their clinical condition had improved (total improvement, partial improvement, no improvement, worsening) and if they were satisfied with the degree of this subjective improvement (satisfied or dissatisfied). Therapeutic success was considered when the patient showed satisfactory subjective improvement.

The objective outcomes assessed were: Visual Analogue Scale (VAS) scores for pain sensation (before and after surgery), scores in the questionnaires NOSE^19^ and SNOT-22,^20^ both validated for the Portuguese language. These outcomes were assessed both quantitatively and qualitatively. The quantitative assessment of the objective outcomes involved statistical calculations comparing pre- and post-topical irrigation therapy.

### Statistical analysis

The results of the scores obtained when applying the Visual Analogue Scale (VAS), NOSE and SNOT-22 questionnaires were described by means, medians and minimum and maximum values. Frequencies and percentages were presented for each of the questions in the questionnaires. The comparison of the groups defined by the treatment (“Xylitol” or “Saline”), in relation to the scores, was carried out using the Mann–Whitney non-parametric test. For the comparison of the moments of assessment (before and after) within each group, the Wilcoxon non-parametric test was considered. Values of *p* < 0.05 indicated statistical significance. The data were analyzed using the computer program Stata / SE v.14.1 (StataCorpLP, USA).

## Results

The analysis presented below was performed based on the responses obtained from 52 patients, of which 26 patients used the xylitol solution (“Xylitol” group) and the other 26 patients used saline solution (“Saline” group) after the surgical procedure.

### Comparison of groups regarding the pain scale (VAS)

Table 2 shows the descriptive statistics of the VAS score and the *p* values of the statistical tests.

**Table 2:** Comparison of groups regarding pain assessment results.

Assessment	Group	VAS score			*p*^a^
		n	Mean	Median (min–max)	
Before	Saline	26	8.2	8 (6–10)	0.779
	Xylitol	26	8.1	8 (6–10)	
After	Saline	26	4.5	5 (2–7)	<0.001
	Xylitol	26	2.6	3 (1–4)	
Reduction	Saline	26	3.7	4 (2–6)	<0.001
	Xylitol	26	5.5	6 (3–7)	

VAS, visual analog scale; max, maximum; min, minimum.

a Mann–Whitney test.

Similar results were obtained regarding pain assessment before the surgery (*p* = 0.779). After the surgical procedure, a significant difference was found between the two groups regarding the pain scale values (*p* < 0.001). Similarly, when comparing the groups regarding the reduction in the VAS score, a significant difference was found between them (*p* < 0.001).

The “Xylitol” group showed an average reduction of 5.5 points, greater than the reduction observed in the “Saline” group (average of 3.7 points). Then, VAS scores before surgery were compared with those after surgery within each group. For both groups, a significant difference was found between the two VAS assessments (“Saline” group: *p* < 0.001 and “Xylitol” group: *p* < 0.001).

### Comparison of groups regarding nasal obstruction symptoms (NOSE)

After the surgical procedure, a significant difference was found between the two groups (*p* < 0.001); (Table 3). The “normal” and “mild” classifications and the “accentuated” and “severe” classifications were grouped. Likewise, when comparing the groups regarding the reduction in the NOSE score, a significant difference was found between them (*p* = 0.002). The “Xylitol” group showed an average reduction of 45.6 points, greater than the reduction in the “Saline” group (average of 31.7 points); (Table 4). Further, NOSE scores were compared within each group, before and after surgery. For this purpose, the null hypothesis that the results would be the same in the two evaluations *versus* the alternative hypothesis of different results was tested. For both groups, a significant difference was found between the two NOSE assessments (“Saline” group: *p* < 0.001 and “Xylitol” group: *p* < 0.001).

**Table 3:** Descriptive results of each of the NOSE questions.

Condition	Assessment	Saline (n = 26)			Xylitol (n = 26)		
		Normal/mild	Moderate	Accentuated/ severe	Normal/mild	Moderate	Accentuated/ severe
Nasal congestion	Before	4 (15.4)	8 (30.8)	14 (53.8)	2 (7.7)	4 (15.4)	20 (76.9)
	After	11 (42.3)	15 (57.7)	−	23 (88.5)	3 (11.5)	−
Nasal obstruction	Before	−	8 (30.8)	18 (69.2)	1 (3.8)	5 (19.2)	20 (76.9)
	After	11 (42.3)	11 (42.3)	4 (15.4)	17 (65.4)	9 (34.6)	−
Difficulty passing air through the nose	Before	3 (11.5)	1 (3.8)	22 (84.6)	2 (7.7)	3 (11.5)	21 (80.8)
	After	14 (53.8)	7 (26.9)	5 (19.2)	22 (84.6)	3 (11.5)	1 (3.8)
Nasal obstruction during sleep	Before	−	2 (7.7)	24 (92.3)	1 (3.8)	5 (19.2)	20 (76.9)
	After	4 (15.4)	19 (73.1)	3 (11.5)	18 (69.2)	8 (30.8)	−
Nasal obstruction during exercise	Before	−	3 (11.5)	23 (88.5)	3 (11.5)	1 (3.8)	22 (84.6)
	After	3 (11.5)	16 (61.5)	7 (26.9)	17 (65.4)	8 (30.8)	1 (3.8)

**Table 4:** Comparative analysis of NOSE scores.

Assessment	Group	NOSE score (0–100)			*p*^a^
		n	Mean	Median (min–max)	
Before	Saline	26	76.7	75 (40–100)	0.921
	Xylitol	26	75.4	80 (25–100)	
After	Saline	26	45.0	45 (15–70)	<0.001
	Xylitol	26	29.8	30 (10–55)	
Reduction	Saline	26	31.7	27.5 (5–65)	0.002
	Xylitol	26	45.6	45 (5–65)	

Max, maximum; min, minimum.

a Mann–Whitney test.

### Comparison of groups in relation to quality of life assessment (SNOT-22)

Table 5, Table 6, present frequencies and percentages of patients according to the treatment (“Saline” or “Xylitol”), for each problem or condition assessed, for the evaluations before and after surgery.

**Table 5:** Descriptive results of each of the SNOT-22 questions (problem).

Problem	Assessment	Saline (n = 26)			Xylitol (n = 26)		
		None/very mild	Mild/slight	Severe/very severe	None/very mild	Mild/slight	Severe/very severe
Need to blow one’s nose	Before	2 (7.7)	19 (73.1)	5 (19.2)	5 (19.2)	17 (65.4)	4 (15.4)
	After	4 (15.4)	22 (84.6)	−	22 (84.6)	4 (15.4)	−
Sneezing	Before	2 (7.7)	21 (80.8)	3 (11.5)	4 (15.4)	20 (76.9)	2 (7.7)
	After	11 (42.3)	15 (57.7)	−	22 (84.6)	4 (15.4)	−
Runny nose	Before	−	21 (80.8)	5 (19.2)	8 (30.8)	16 (61.5)	2 (7.7)
	After	12 (46.2)	14 (53.8)	−	19 (73.1)	7 (26.9)	−
Cough	Before	4 (15.4)	11 (42.3)	11 (42.3)	6 (23.1)	7 (26.9)	13 (50)
	After	7 (26.9)	17 (65.4)	2 (7.7)	11 (42.3)	15 (57.7)	−
Sensation of secretion	Before	2 (7.7)	8 (30.8)	16 (61.5)	4 (15.4)	8 (30.8)	14 (53.8)
	After	13 (50)	12 (46.2)	1 (3.8)	18 (69.2)	8 (30.8)	−
Thick phlegm in the nose	Before	1 (3.8)	20 (76.9)	5 (19.2)	6 (23.1)	16 (61.5)	4 (15.4)
	After	18 (69.2)	8 (30.8)	−	22 (84.6)	4 (15.4)	−
Muffling in the ear	Before	11 (42.3)	12 (46.2)	3 (11.5)	8 (30.8)	15 (57.7)	3 (11.5)
	After	21 (80.8)	5 (19.2)	−	26 (100)	−	−
Dizziness	Before	24 (92.3)	1 (3.8)	1 (3.8)	20 (76.9)	5 (19.2)	1 (3.8)
	After	24 (92.3)	1 (3.8)	1 (3.8)	26 (100)	−	−
Earache	Before	11 (42.3)	13 (50)	2 (7.7)	19 (73.1)	6 (23.1)	1 (3.8)
	After	23 (88.5)	3 (11.5)	−	25 (96.2)	1 (3.8)	−
Facial pain or pressure	Before	2 (7.7)	10 (38.5)	14 (53.8)	5 (19.2)	1 (3.8)	20 (76.9)
	After	11 (42.3)	15 (57.7)	−	8 (30.8)	18 (69.2)	−
Difficulty falling asleep	Before	10 (38.5)	8 (30.8)	8 (30.8)	4 (15.4)	11 (42.3)	11 (42.3)
	After	14 (53.8)	7 (26.9)	5 (19.2)	8 (30.8)	17 (65.4)	1 (3.8)
Waking up in the middle of the night	Before	10 (38.5)	5 (19.2)	11 (42.3)	3 (11.5)	20 (76.9)	3 (11.5)
	After	13 (50)	11 (42.3)	2 (7.7)	12 (46.2)	14 (53.8)	−

**Table 6:** Descriptive results of each of the SNOT-22 questions.

Problem	Assessment	Saline (n = 26)			Xylitol (n = 26)		
		None/very mild	Mild/slight	Severe/very severe	None/very mild	Mild/slight	Severe/very severe
Lack of a good night's sleep	Before	9 (34.6)	10 (38.5)	7 (26.9)	4 (15.4)	18 (69.2)	4 (15.4)
	After	12 (46.2)	12 (46.2)	2 (7.7)	18 (69.2)	7 (26.9)	1 (3.8)
Waking up tired in the morning	Before	6 (23.1)	11 (42.3)	9 (34.6)	6 (23.1)	17 (65.4)	3 (11.5)
	After	13 (50)	11 (42.3)	2 (7.7)	24 (92.3)	2 (7.7)	−
Tiredness/fatigue throughout the day	Before	6 (23.1)	11 (42.3)	9 (34.6)	6 (23.1)	16 (61.5)	4 (15.4)
	After	15 (57.7)	9 (34.6)	2 (7.7)	23 (88.5)	3 (11.5)	−
Decreased productivity	Before	11 (42.3)	11 (42.3)	4 (15.4)	9 (34.6)	14 (53.8)	3 (11.5)
	After	17 (65.4)	9 (34.6)	−	25 (96.2)	1 (3.8)	−
Decreased concentration	Before	10 (38.5)	14 (53.8)	2 (7.7)	11 (42.3)	12 (46.2)	3 (11.5)
	After	17 (65.4)	9 (34.6)	−	24 (92.3)	2 (7.7)	−
Frustrated	Before	13 (50)	9 (34.6)	4 (15.4)	14 (53.8)	9 (34.6)	3 (11.5)
	After	19 (73.1)	7 (26.9)	−	24 (92.3)	2 (7.7)	−
Sad	Before	21 (80.8)	2 (7.7)	3 (11.5)	21 (80.8)	2 (7.7)	3 (11.5)
	After	23 (88.5)	3 (11.5)	−	26 (100)	−	−
Embarrassed	Before	18 (69.2)	5 (19.2)	3 (11.5)	22 (84.6)	2 (7.7)	2 (7.7)
	After	25 (96.2)	1 (3.8)	−	25 (96.2)	1 (3.8)	−
Perception of smell or taste	Before	5 (19.2)	9 (34.6)	12 (46.2)	3 (11.5)	2 (7.7)	21 (80.8)
	After	7 (26.9)	19 (73.1)	−	7 (26.9)	18 (69.2)	1 (3.8)
Stuffy nose	Before	2 (7.7)	2 (7.7)	22 (84.6)	2 (7.7)	3 (11.5)	21 (80.8)
	After	2 (7.7)	24 (92.3)	−	8 (30.8)	18 (69.2)	−

The classifications “None/Very mild”, “Mild/Slight” and “Moderate” and “Severe/Very severe” were grouped.

The results indicate homogeneous groups in the assessment of quality of life by SNOT-22 before the surgery (*p* = 0.863). After the surgery, a significant difference was found between them (*p* < 0.001). Likewise, when comparing the groups regarding the reduction in the SNOT-22 score, a significant difference was found between them (*p* = 0.001). The “Xylitol” group showed an average reduction of 32.9 points, greater than the reduction for the “Saline” group (average of 21.3 points).

Then, within each group, SNOT-22 scores before surgery were compared with those after surgery. For both groups, a significant difference was found between the two SNOT-22 assessments (“Saline” group: *p* < 0.001; and “Xylitol” group: *p* < 0.001); (Table 7).

**Table 7:** Comparative analysis of SNOT-22 scores.

Assessment	Group	SNOT-22 score (0–110)			*p*^a^
		n	Mean	Median (min–max)	
Before	Saline	26	51.8	50 (17–101)	0.863
	Xylitol	26	51.7	53.5 (13–110)	
After	Saline	26	30.5	29.5 (11–52)	< 0.001
	Xylitol	26	18.8	19 (2–34)	
Reduction	Saline	26	21.3	20 (6–60)	0.001
	Xylitol	26	32.9	31.5 (10–99)	

Max, maximum; min, minimum.

a Mann–Whitney test.

## Discussion

The present prospective study showed that the group of patients who used xylitol solution in the postoperative period of endonasal endoscopic surgery showed significant improvement in pain symptoms (through the visual analog scale assessment) and nasal symptoms (through scores in the NOSE and SNOT-22 questionnaires). Weissman et al. demonstrated the efficacy of xylitol irrigation in improving nasal symptoms in patients with CRS.^17^ However, the treatment was carried out for only 10 days. Lin et al. also demonstrated the superiority of nasal irrigation with xylitol over saline solution in patients with CRS. The xylitol solution was prepared at a concentration of 5% and both groups used their respective solutions for a period of 30 days.^18^

Xylitol is a non-toxic and well-tolerated substance, with safe daily doses, without previously established adverse effects.^5^, ^21^ Xylitol has several therapeutic effects, affecting the growth of *Streptococcus pneumoniae* and *Haemophilus influenzae*^7^, ^22^ and bacterial adhesion,^10^, ^22^, ^23^, ^24^ increased innate immunity,^13^ increased nitric oxide production^15^, ^25^ and biofilm formation inhibition.^16^, ^26^ Ammons et al.^26^, ^27^ (in 2009 and 2011) conducted several studies in which they demonstrated the ability of xylitol to dissolve the structure of *Pseudomonas aeruginosa* biofilm. This bacterial biofilm has a high clinical relevance in humans (highly prevalent in the cases of CRS) and it is one of the most resistant to common antimicrobials.^26^ In a previous study, Katsuyama et al. had already demonstrated the effectiveness of xylitol (at a concentration of 5%) in inhibiting the formation of *Staphylococcus aureus* biofilm, mainly by inhibiting the formation of the glycocalyx.^28^

Several experimental studies have shown the beneficial potential of xylitol, such as reduced adherence of *S. pneumoniae in vitro*,^29^ reduction in biofilm formation by the pneumococcus,^30^ and reduction in the concentration of *P. aeruginosa* in the maxillary sinuses of inoculated rabbits.^2^ The therapeutic effects of xylitol on the airways can be identified within short periods of time. Zabner et al.^13^ demonstrated an increase in the activity of the immune system in the nasal mucosa of 21 healthy subjects who used xylitol solution for only four days.^15^ Similarly, Weissman et al. and Lin et al. observed satisfactory results with short periods of use of the xylitol nasal solution.^17^, ^18^ This characteristic strengthens our belief that xylitol solution can be recommended and prescribed for a short period of time, increasing patient compliance and quality of life. In the present study, the choice of the time interval for using the xylitol solution (30 days) was made at random. We believe, however, that further studies to assess the ideal minimum time interval for obtaining the highest degree of patient satisfaction are still required.

Several other forms of nasal irrigation have been proposed for the same purpose. Giotakis et al. randomized the use of saline solution among 174 subjects submitted to FESS and observed a significant improvement in nasal symptoms in the subjects from the group that made systematic use of nasal irrigation (0.9% saline solution only).^31^ More interestingly, they observed that after 3 months of nasal irrigation, the beneficial effects were no longer different between the two groups.^31^ Kosugi et al. obtained significant improvement in 16 subjects instructed to perform nasal irrigation with high-volume budesonide (1 mg of budesonide diluted in 500 mL of saline solution every two days), in the scores of the SNOT-22 questionnaire and Lund-Kennedy endoscopic classification, after 3 months of therapy.^32^ The study by Low et al. evaluated the symptoms and mucociliary clearance in 74 adult subjects submitted to FESS and were instructed to perform nasal irrigation with saline solution (0.9%), lactated Ringer or hypertonic saline solution (2.7%). They observed that all groups showed improvement in clinical scores (SNOT-20 and VAS) and in the endoscopic aspect of the nasal mucosa after the sixth postoperative week with nasal irrigation, but without showing an impact on mucociliary clearance improvement. They also observed a significant improvement in symptoms with the use of lactated Ringer solution.^33^ Xylitol, used in the present study, was effective in significantly reducing adverse nasal symptoms (as observed by the scores in the NOSE and SNOT-22 questionnaires).

Based on previous studies, one can speculate that the improvement in patients' symptoms would be related to the improvement in the mechanisms associated with the pathophysiology of CRS itself (by improving mucociliary clearance and reducing biofilm formation) and also to the improvement of postoperative healing. The healing of the nasal mucosa, after surgical trauma, involves a complex process aimed to reestablishing the anatomical and functional integrity of the nasal and paranasal cavities.^34^ Several topical solutions have already been tested *in vitro* and *in vivo* (in animal models) aiming to accelerate the healing process, as well as in the clinical treatment of CRS.^34^ Nasal irrigation is useful in the treatment of rhinosinusitis and in postoperative nasal surgeries, as it removes secretions and debris, minimizing the formation of crusts and synechia and improving mucociliary transport.^35^, ^36^ Nasal lavage is usually performed with 0.9% isotonic saline solution. However, studies carried out with hypertonic saline solutions have reported that these substances alter the osmotic pressure and thus reduce edema, improving mucociliary clearance.^37^

### Limitations

The present study had some limitations. First, the sample size was small. Although we included 52 patients, other studies available in the literature involved almost 200 participants. Second, the period of time during which the subjects included in the present study had CRS symptoms may have had some influence on the results obtained. In other words, a subject diagnosed with CRS for a longer time may have had less therapeutic success with nasal irrigation with xylitol. Third, factors external to the research could have had an effect on the results. These include, for instance, professional activity, place of residence, type of residence, associated comorbidities, other medications being used, among others. Another limitation is that the effect of xylitol on nasal symptoms was assessed within 30 days after endonasal endoscopic surgery. Other assumptions about the long-term effect of xylitol on nasal symptoms cannot be made. Finally, the present study used subjective symptom assessment questions (answered by the subjects included in the study). The lack of objective parameters (tomographic comparisons before and after surgery and nasal irrigations, for instance) could be considered a bias in the present study. However, it is noteworthy that postoperative imaging exams are not reimbursed by health plans in Brazil. As it would be an ethical infraction to financially burden the subjects included in the study, we decided not to perform the radiological procedure after the analyzed period.

## Conclusion

The present study showed that the use of xylitol in nasal irrigations in the postoperative period of FESS significantly reduced the nasal symptoms of the assessed subjects. Xylitol can be used in the postoperative period of FESS without causing side effects, as it leads to greater reduction in nasal symptoms. Future studies are required with a larger sample size, randomized as to the type of nasal irrigation and with objective measures by means of imaging tests, for adequate control of CRS.

## Conflicts of interest

The authors declare no conflicts of interest.
